# Analysis of Nanoscratch Mechanism of C-Plane Sapphire with the Aid of Molecular Dynamics Simulation of Hcp Crystal

**DOI:** 10.3390/nano11071739

**Published:** 2021-07-01

**Authors:** Wangpiao Lin, Naohiko Yano, Jun Shimizu, Libo Zhou, Teppei Onuki, Hirotaka Ojima

**Affiliations:** 1 Graduate School of Science and Engineering, Ibaraki University, 4-12-1 Nakanarusawa-cho, Hitachi-shi, Ibaraki 316-8511, Japan; lwp511430783@163.com (W.L.); junshim01@gmail.com (N.Y.); 2 Department of Mechanical Systems Engineering, Ibaraki University, 4-12-1 Nakanarusawa-cho, Hitachi-shi, Ibaraki 316-8511, Japan; libo.zhou.1618@vc.ibaraki.ac.jp (L.Z.); teppei.onuki.nlab@vc.ibaraki.ac.jp (T.O.); hirotaka.ojima.gen365@vc.ibaraki.ac.jp (H.O.)

**Keywords:** nanoscratch, molecular dynamics simulation, sapphire, hcp crystal, basal slip

## Abstract

In this study, single groove nanoscratch experiments using a friction force microscope (FFM) with a monocrystalline diamond tip were conducted on a c-plane sapphire wafer to analyze the ductile-regime removal and deformation mechanism including the anisotropy. Various characteristics, such as scratch force, depth, and specific energy for each representative scratch direction on the c-plane of sapphire, were manifested by the FFM, and the results of the specific scratch energy showed a trend of six-fold symmetry on taking lower values than those of the other scratch directions when the scratch directions correspond to the basal slip directions as $\left(0001\right)\langle 11\bar{2}0\rangle$. Since this can be due to the effect of most probably basal slip or less probably basal twinning on the c-plane, a molecular dynamics (MD) simulation of zinc, which is one of the hexagonal close-packed (hcp) crystals with similar slip/twining systems, was attempted to clarify the phenomena. The comparison results between the nanoscratch experiment and the MD simulation revealed that both the specific scratch energy and the burr height were minimized when scratched in the direction of the basal slip. Therefore, it was found that both the machining efficiency and the accuracy could be improved by scratching in the direction of the basal slip in the single groove nanoscratch of c-plane sapphire.

## 1. Introduction

Single crystal sapphire (α-Al_2_O_3_) is widely used in various industries due to superior physical, chemical and optical properties, namely c-plane sapphire substrates are widely used to grow group III-V and II-VI element-based semiconductor compounds such as GaN for blue LEDs and laser diodes [1,2]. Therefore, it is important to understand the material removal mechanism during the manufacturing process of sapphire wafer, especially in the nanoscale process [3]. However, sapphire exhibits anisotropy which directly affects the elastic-plastic deformation mechanism and depends on orientations during machining process [4,5,6].

Recently, extensive investigations were conducted to reveal deformation behavior concerning anisotropy of sapphire [7]. Hockey [8] and Chan, et al. [9] pointed out the twin system of basal and rhombohedral planes through static indentation of sapphire. Lin, et al. [10] based their study on the molecular dynamic method to investigate deformation of c-plane sapphire under nanoindentation. Although comprehensive research has been carried out to study elastic-plastic deformation behavior in the static state by indentation, few studies have concentrated on sapphire anisotropy under the dynamic state [11,12]. Mizumoto, et al. [13] discussed the anisotropic deformation behavior of monocrystalline sapphire by plunge-cut tests in brittle–ductile transition. Nanoscratch is becoming a promising characterization method to analyze wear behavior and the nano-tribological property of a material under the dynamic state [14,15]. However, there are few studies focused on the machining anisotropy of monocrystalline sapphire in the ductile-regime by nanoscratch or similar methods which have clarified particularly the effect of the tool rake angle [16] and the slip/twinning systems [17] on the deformation states—even though it is important to clarify the deformation and removal mechanisms including the anisotropy, which are particularly concerned with the machining efficiency and machined surface and sub-surface qualities.

In this study, in order to clarify the ductile-regime machining mechanism of c-plane sapphire including its anisotropy, particularly, in order to clarify the scratch direction that is efficient for material removal rate and/or machined surface accuracy—because they have already not been clarified—a series of single groove nanoscratch experiments using a friction force microscope (FFM) with a monocrystalline diamond tip was conducted on c-plane sapphire wafer substrate. Particularly, the effects of the scratch direction on scratch force, groove depth, and specific energy were examined by FFM. Then, molecular dynamics (MD) simulation was applied to zinc (Zn), one of the hexagonal close-packed (hcp) crystals with a similar slip/twinning system, to compare with the experimental results and to clarify the similarity with the deformation mechanism of hcp crystals as well as the ductile-regime machining mechanism of c-plane sapphire wafer through single groove nanoscratch.

## 2. Crystal Structure and Anisotropy

### 2.1. Structure Feature

**Figure 1:**
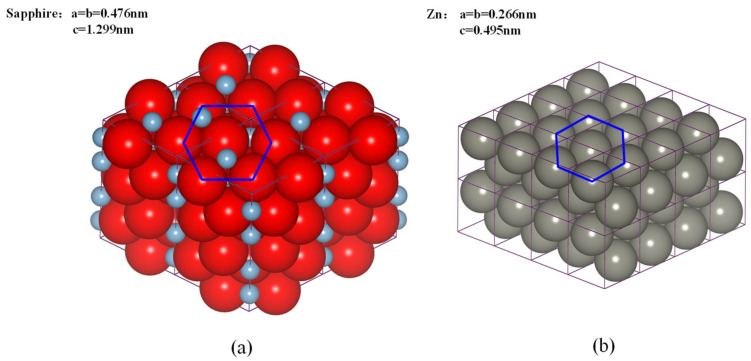
Schematic of the crystal arrangement of (**a**) sapphire (blue and red balls represent Al^3+^ and O^2−^ ions, respectively.) and (**b**) Zn, respectively.

Although single crystal sapphire originally has a rhombohedral structure, it is often approximated by an hcp crystal for convenience. To clearly reveal the anisotropic characteristics of single crystal sapphire, it is essential to display the crystal structure of sapphire. Figure 1 displays the structure of (a) single crystal sapphire compared to (b) Zn which was used in the MD simulation. The crystal structure of sapphire consists of oxygen ions closely packed with aluminum ions embedded in oxygen interstices [18]. According to the Bravais classification of a symmetry crystal, the structure of monocrystalline sapphire belongs to the space group as shown in Figure 1a, and can be described as either a hexagonal or rhombohedral unit cell [19]. On the other hand, Zn with a similar hcp structure [20] belongs to the P6_3_/mmc space group. In Figure 1, it is clear to see the similar hexagonal structure of sapphire and Zn in the basal plane.

### 2.2. Anisotropic Properties

To illustrate the deformation mechanism of monocrystalline sapphire during nanoscratch, the possible slip/twinning systems and critical shear stresses of sapphire crystal were listed as in Table 1 [14,19,21]. As shown in Table 1, critical shear stress varies with orientation and thus slip or twinning is more likely to occur on the crystal plane where the shear stress exceeds the critical shear stress. Table 2 shows the representative slip/twin systems for Zn [22] for comparison. Comparing Table 1 and Table 2, it can be seen that the basal slip system is totally the same and the primary twinning system is also similar to the rhombohedral and tensile ones, respectively.

**Figure 2:**
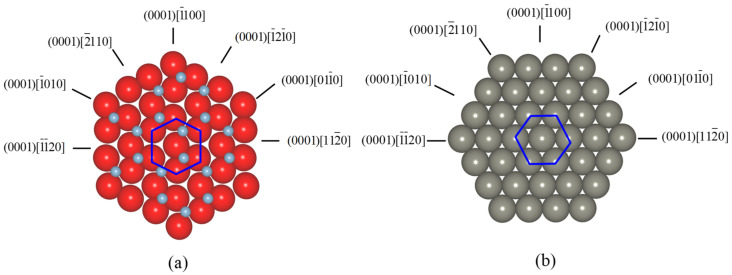
Top view of basal slip or twinning directions of (**a**) sapphire and (**b**) Zn.

Figure 2 shows the top view of basal slip or twinning directions of (a) sapphire and (b) Zn, respectively. The same structure of six-fold symmetry of sapphire and Zn can obviously be seen in the basal plane. According to the critical shear stress shown in Table 1, the possibility of twinning deformation on the rhombohedral plane is relatively high. However, what is the most important is the resolved shear stress exerted on the possible slip/twin systems when a scratching force is applied. It should be noted that in sapphire, basal slip and twinning and prism and pyramidal slips may show 6-fold symmetry, while rhombohedral twinning may show 3-fold symmetry according to the crystal structure.

## 3. Experimental Set-Up for Nanoscratch

**Figure 3:**
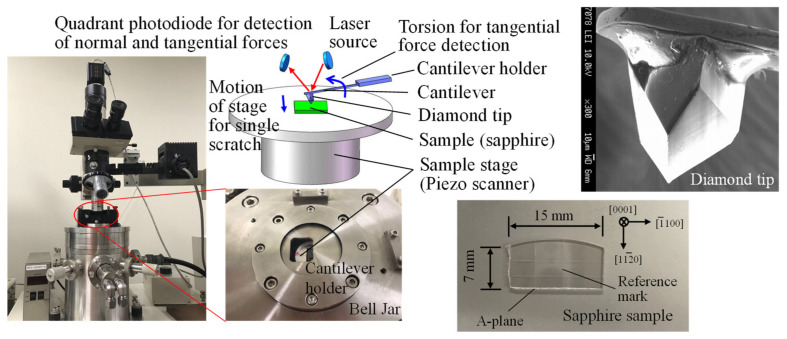
Appearance of SPM and schematic of nanoscratching model using FFM mode with the images of sapphire sample and diamond tip.

Nanoscratch tests were performed on an SPM (SPA-300HV, Seiko Instruments Co., Japan) with its FFM function using a monocrystalline diamond tip as the scratch tool as shown in Figure 3. During the nanoscratch, a constant normal load was given through bending of a cantilever on which a diamond tip was bonded and monitored and controlled by a piezo scanner/actuator, while the scratch force was monitored using the cantilever’s torsion (torsional displacement). This was detected through a quadrant photodiode considering the torsional stiffness of the cantilever. A 2-inch double-sided polished epi-ready c-plane (0001) sapphire wafer substrate with an A-plane cross-section was cut into a small piece and used as the workpiece as shown in Figure 3.

**Figure 4:**
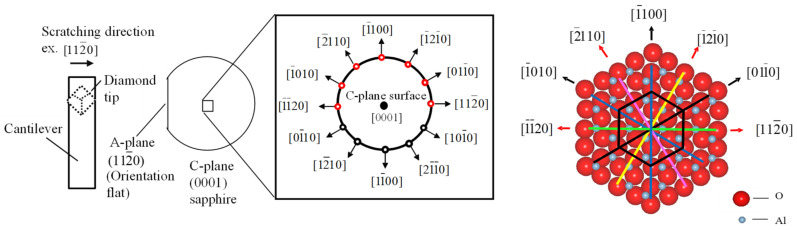
Schematic of defined nanoscratch directions on c-plane sapphire.

The scratch directions on the c-plane sapphire are shown in Figure 4. The anisotropy of machinability was investigated by scratching in seven different directions every 30 degrees as shown in Figure 4. The groove geometries were directly measured on the SPM using the same diamond tip used for nanoscratch immediately after each single groove nanoscratch test.

## 4. Experimental Results

### Scratch Force, Depth, and Specific Energy

The nanoscratch experiments were conducted on a c-plane sapphire workpiece. A series of experimental conditions is listed in Table 3.

**Figure 5:**
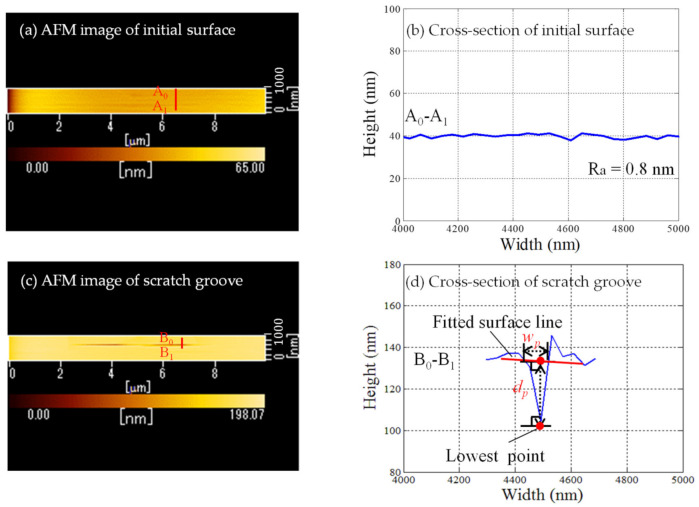
AFM images and cross-sections of initial surface (**a**,**b**) and scratch groove (**c**,**d**), respectively.

Figure 5 shows AFM images (a) and cross-sections of initial surface (b), scratch groove (c), (d), respectively, where the normal load is 10 µN and the scratch direction is $\left[11\bar{2}0\right]\;$in Figure 5c,d. After each trial, scratch force (tangential force) and depth of scratch grooves were directly measured by the AFM mode of the SPM. Scratch depth and width for each scratch direction was determined using the method shown in Figure 5d and averaged from the results of the five cross-sections.

**Figure 6:**
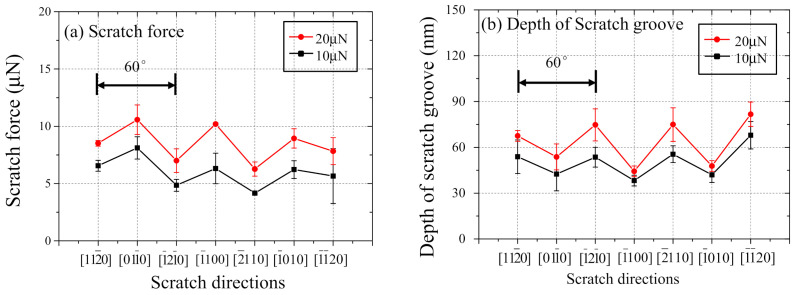
The relationship between scratch direction and (**a**) scratch force and (**b**) depth of scratch groove, respectively.

Figure 6 shows the relationship between scratch direction and (a) scratch force and (b) groove depth, respectively. Publications [23,24,25] also reported that the critical depth of cut for sapphire in the ductile-regime was less than 0.24 µm while nanoscratched depths were lower than 85 nm in this study. Therefore, the ductile-regime removal and deformation mechanisms should be discussed in this study, because all the nanoscratch experiments were completed within the ductile-regime.

In Figure 6a, higher scratch forces are obtained when the scratch directions are, $\left[01\bar{1}0\right]$, $\left[\bar{1}100\right],$ and $\left[\bar{1}010\right]$ than those of $\left[11\bar{2}0\right]$, $\left[\bar{1}2\bar{1}0\right]$, $\left[\bar{2}110\right],$ and $\left[\bar{11}20\right]$. On the contrary, Figure 6b indicates that deeper groove depths are observed when the scratch directions are $\left[11\bar{2}0\right]$, $\left[\bar{1}2\bar{1}0\right]$, $\left[\bar{2}110\right],$ and $\left[\bar{11}20\right]$ than those of $\left[01\bar{1}0\right]$, $\left[\bar{1}100\right],$ and $\left[\bar{1}010\right]$. According to both figures, the intervals between adjacent peaks or valleys are 60°, such as the $\left[01\bar{1}0\right]$ and $\left[\bar{1}100\right]$ directions. It illustrates that c-plane sapphire has six-fold symmetrical machinability originating from the atomic arrangements in the basal plane. On the other hand, the rhombohedral planes have three-fold symmetry. Therefore, the influence of the rhombohedral twinning on the deformation would be expected to be small.

**Figure 7:**
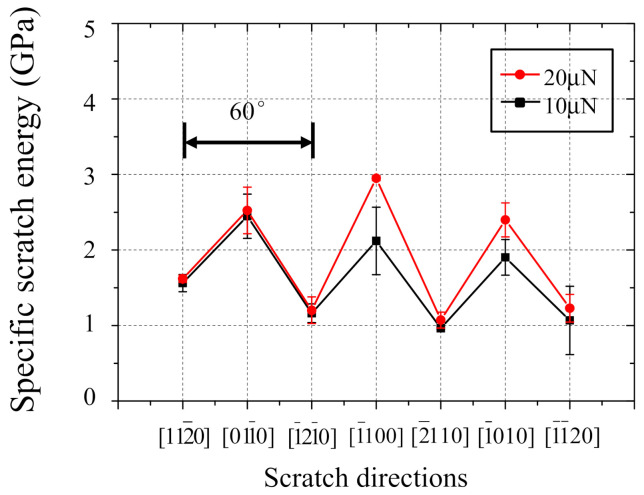
The relationship between scratch direction and specific scratch energy.

To more accurately grasp the anisotropy in machinability, the relationship between scratch direction and specific scratch energy is shown in Figure 7. The specific scratch energy is calculated considering the cross-sectional shape of the scratch groove (a triangle as shown in Figure 5d) using the following formula:

(1) $$E=\frac{F_{t}L}{v}=\frac{2F_{t}}{d_{p}w_{p}}$$

where *E* is specific scratch energy, *F_t_* is scratch (tangential) force, *L* is scratch length (5 µm), *v* is scratched volume, *d_p_* is depth of scratch groove, and *w_p_* is groove width.

When the scratch directions are $\left[11\bar{2}0\right]$, $\left[\bar{1}2\bar{1}0\right]$, $\left[\bar{2}110\right],$ and $\left[\bar{11}20\right]$, the lower specific scratch energies are observed. The specific scratch energies are around 1 GPa which is far less than those of the $\left[01\bar{1}0\right]$, $\left[\bar{1}100\right],$ and $\left[\bar{1}010\right]$ directions. The specific scratch energy clearly shows six-fold symmetry as seen in Figure 6a,b. It also revealed that the basal plane played an essential role in the material deformation of sapphire being different from the prism, pyramidal, and rhombohedral planes when single groove nanoscratch is performed on c-plane sapphire. Furthermore, it also implied that the lower specific scratch energies were observed along the basal slip directions, and it indicates higher possibility of basal slip to occur than that of basal twinning.

## 5. Molecular Dynamics Modeling for Hcp Crystal

The MD simulation is visualized and an effective method for understanding the material removal and deformation mechanisms in nanoscale machining processes [26,27]. In order to reveal that the deformation on the basal plane plays an essential role for the hcp crystal when nanoscratch tests are conducted on c-plane substrate, in this study, MD simulations of Zn, one of the simple hcp crystals, were performed to basically understand the fundamental removal and deformation mechanisms, since sapphire has a more complicated atomic structure than Zn.

**Figure 8:**
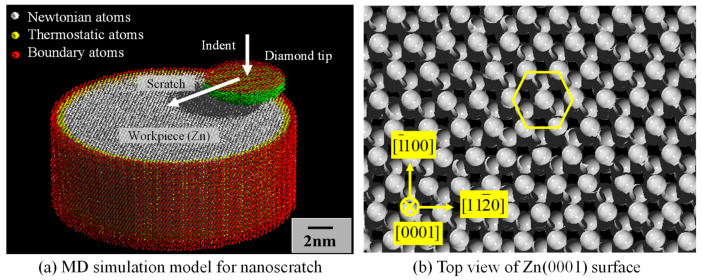
(**a**) MD simulation model for nanoscratch and (**b**) top view of Zn(0001) surface.

The self-developed MD simulation model for nanoscratch tests is shown in Figure 8a. The workpiece and the tool tip are assumed to consist of monocrystalline Zn and diamond (C), respectively. In the MD simulation [28], by taking the mass of atom *i* to be *m_i_*, its position at time *t* to be ***R****_i_*(*t*), and the resultant force obtained by summing up the interactions from all the neighboring atoms to be ***F_i_***(*t*), the following equation of motion of Newton’s second law is obtained:

(2) $$m_{i}\frac{d^{2}R_{i}\left(t\right)}{dt^{2}}=F_{i}\left(t\right)$$

The interatomic forces can be calculated from the gradient of the interatomic potential functions explained later. Equation (2) is applied to all the atoms of interest and solved using the leap-frog method [29] and solved individually at each time step, where the time step is 2 fs. In the present trials, Morse potential functions were used for Zn–Zn [30] and Zn–C interactions with the parameters shown in Table 4, respectively. If emphasis is placed on the characterization of Zn, an EAM (embedded-atom method) potential [31] should be used. However, since the comparison target in this paper is sapphire, a hard and brittle material, for the purpose of investigating the similarity in the deformation with hcp crystals, a Morse potential, that tends to underestimate the ductility, was used between a pair of Zn–Zn atoms.

Morse potential function is as follows:

(3) $$\emptyset \left(r_{ij}\right)=D\left[\exp\left\{-2\alpha \left(r_{ij}-r_{0}\right)\right\}-2\exp\left\{-\alpha \left(r_{ij}-r_{0}\right)\right\}\right]$$

where ∅ is the potential energy, *r_ij_* is the interatomic distance between *i* and *j* atoms, *D* is the dissociation (cohesion) energy, *r*_0_ is the equilibrium bond length, and α is the potential coefficient, respectively.

Since the potential parameters between Zn and C are unknown, they were estimated using the mixing law [32], and *D* was reduced to 1/10 to prevent non-physical phenomena due to extremely strong adhesion between the workpiece and the diamond tip. It was considered more realistic to treat it as such in the case of the actual scratching events in the atmosphere. The cut off distance was set to 3*a* here.

The Tersoff potential function [33], which is one of the most popular multibody interatomic potentials for diamond, can be applied to the interactions among C atoms of the diamond tip [34,35]. Generally, the total energy *E_s_* of the system in the form of the Tersoff potential is as follows:

(4) $$E_{s}=\frac{1}{2}\sum_{i}\sum_{j\neq i}f_{C}\left(r_{ij}\right)\left\{a_{ij}f_{R}\left(r_{ij}\right)+b_{ij}f_{A}\left(r_{ij}\right)\right\}$$

where $f_{C}\left(r_{ij}\right)$ is the cutoff function, $f_{R}\left(r_{ij}\right)$ is the repulsive force term, $f_{A}\left(r_{ij}\right)$ is the attractive force term, $b_{ij}$ is the multi-body force term and $a_{ij}$ is taken as 1, generally.

For simplification, all the trials were performed in vacuum, and no chemical reactions were considered. The workpiece was assumed to have a free, completely clean and ideal Zn(0001) structure in its uppermost surface as shown in Figure 8b. Other than the up-permost surface of Zn was surrounded by a thermostat layer (Nose-Hoover thermostat using the velocity scaling method to keep temperature constant [29]) where atoms are arrayed in a lattice constant length *a* of Zn. The mechanical energies increased in the analysis area due to the plastic deformations by scratching being able to be dissipated from this thermostat layer. The area outside the thermostat layer was assumed to be a perfect rigid body. The diamond tip was also composed of the analysis area, thermostat, and perfect rigid body layers. Such a model is often used in the MD simulation of machining processes. A cylindrical workpiece is used because a cubic workpiece is not suitable for investigating the anisotropy of machinability. Since the depths of the scratch grooves were smaller than the diamond tip radius in the experiment, the shape of the tip was assumed to be hemispherical in the simulation as well. Due to the limited size of the model, it was difficult to completely eliminate the effect of the boundaries on the deformation. Nevertheless, the developed model was large enough to analyze the machining anisotropy due to crystal anisotropy.

In the preparation of the initial MD model, considering the principle of minimum potential energy, atomic arrays of Zn and diamond were obtained at absolute zero temperature, then the atomic arrays at 300 K for the workpiece and diamond tip were arranged considering the thermal expansion, and mean-velocity vectors at 300 K, randomly given to all atoms. Next, Newton’s motion-equations were solved and velocity scaling was implemented sequentially until the system became stable, when the analysis area and the thermostat layer were calculated separately. As a result, atomic array models with surfaces were constructed. It was also confirmed that the velocity of the atoms followed Maxwell distributions. These results confirmed the validity of the initial array models. At this stage, we had an MD simulation of the microcanonical ensemble, where the number of atoms, temperature, and energy were both constant. On the other hand, after the motion of the diamond tip has started, it is a non-equilibrium MD simulation, where the number of atoms is constant and only the temperature of the thermostat is constant (300 K).

## 6. MD Simulation Results and Discussion

### 6.1. MD Simulation Results

The series of the simulation conditions is listed in Table 5. A diamond tip was slid horizontally onto the surface of the workpiece at a constant speed of 20 m/s by giving a displacement equivalent to 20 m/s at each time step after indenting it into the Zn(0001) surface to a depth of 1.3 nm. The scratch directions were chosen referencing those of the experiments. Although 20 m/s is remarkably faster than that of the experiment, it is slow enough compared to the speed of elastic and plastic waves, and it can be a reasonable speed to evaluate the plastic deformations of the crystal. Also, the results of scratching at the speed lower than single digits were almost identical. Since constant load control may lead to vibration of the diamond tip, for stability, simulations with a constant depth of cut were attempted, being different from the experiments.

**Figure 9:**
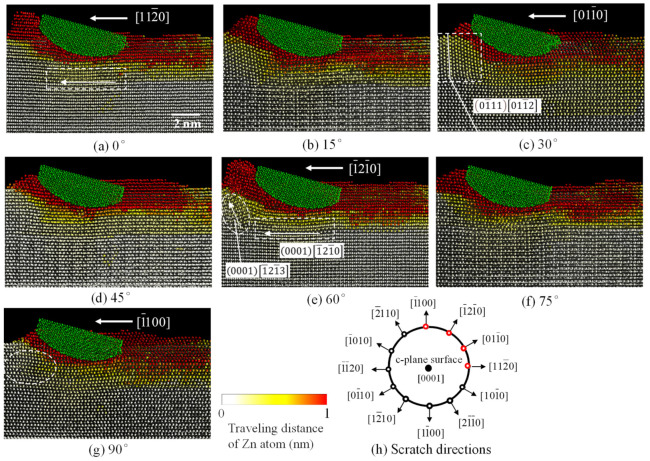
Snapshots of the cross-section for various scratch directions such as (**a**) 0, (**b**) 15, (**c**) 30, (**d**) 45, (**e**) 60, (**f**) 75 and (**g**) 90 degrees from $\left[11\bar{2}0\right]$, where the color bar shows the traveling distance of Zn atom from initial position, and (**h**) is the schematic for scratch directions (MD).

Figure 9 shows the snapshots of the cross-section for various scratch directions, where each cross-section is parallel to the scratch direction of interest. In Figure 9, (a) to (g) show the results when the $\left[11\bar{2}0\right]$ direction is set to be (a) 0 degree and the scratch direction is tilted every 15 degrees in the counterclockwise direction, and (h) shows the schematic of the scratch directions. In the case of (a) $\left[11\bar{2}0\right]$ (0 deg.) and (e) $\left[2\bar{11}0\right]$ (60 deg.) directions, short cutting chips are generated, respectively, and remarkably similar forms of deformation can be observed. The Zn atoms surrounded by the square beneath the diamond tip slide horizontally to the left, such as along $\left(0001\right)\left[11\bar{2}0\right]$ and $\left(0001\right)\left[\bar{1}2\bar{1}0\right],\;$and cause basal slip deformations. The basal slips change their directions diagonally upward at the region diagonally below and forward of the diamond tip as circled by the ellipse in Figure 9e. This plays a major role for the formation of the shear planes in a cutting chip. In particular, basal slip is important in promoting chip generation because its deformation capacity is larger than that of twinning. From Figure 9e, it can be seen that the deformation diagonally upward after the basal slip is complicated with a mixture of pyramidal slips <*c*+*a*> along $\left(\bar{1}2\bar{1}3\right)\left[1\bar{2}12\right]\;$and partial dislocations. This is considered to be less deformable than the pure slip represented by the basal one. In the case of sapphire, the effects of pyramidal slip, rhombohedral twinning, and partial dislocations are expected to be mixed in a complex manner.

On the other hand, in the case of (c) $\left[10\bar{1}0\right]$ (30 deg.) and (g) $\left[\bar{1}100\right]$ (90 deg.), they are limited to occurrence of wedge-formation type ploughing rather than the formation of short cutting chips. The deformations of (c) and (g) are also similar. From Figure 9e, it can be observed that the region surrounded by a square has a different crystal arrangement from the deeper parts (see the solid lines). This is due to the compression twin along $\left(0\bar{1}11\right)\left[01\bar{1}2\right]$ based on partial dislocations. A kind of partial dislocation is also found in the region shown by the ellipse in Figure 9g. In the case of sapphire, basal twinning is expected to occur.

In the case of intermediate (b) 15 deg., (d) 45 deg. and (f) 75 deg., it can be seen that the chip formation heights are intermediate of the former different two groups.

**Figure 10:**
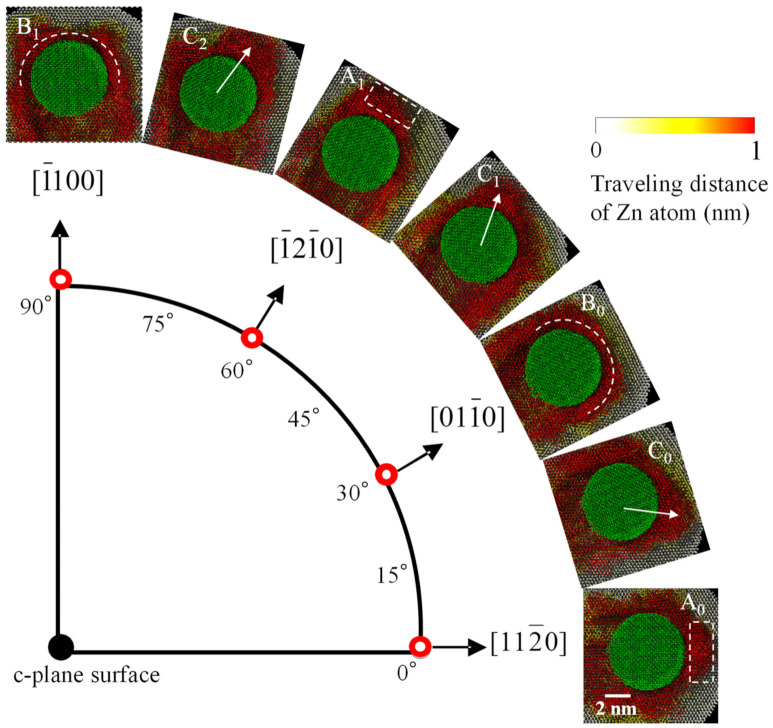
Top views in all the scratch directions to observe the chip formation morphologies, where the color bar shows the traveling distance of Zn atom from initial position (MD).

Figure 10 is the result of displaying the top views in all the scratch directions to observe the chip formation morphologies. From the result, it can be observed that the cutting chips are smoothly generated forward when scratched in the basal slip direction (see, rectangles of A_0_ and A_1_). On the other hand, it can be observed that burrs are likely to occur in the lateral direction when scratched in the 30 and 90 degrees directions (see, half circles of B_0_ and B_1_). These trends are almost the same as that of the MD simulation results obtained by Lin, et al. [17]. Furthermore, it can be also observed when scratched in the 15, 45 and 75 deg. directions, the cutting chips are generated tilting toward the basal slip directions (see, arrows of C_0_, C_1,_ and C_2_). These results also clearly show that the basal slip is highly deformable.

**Figure 11:**
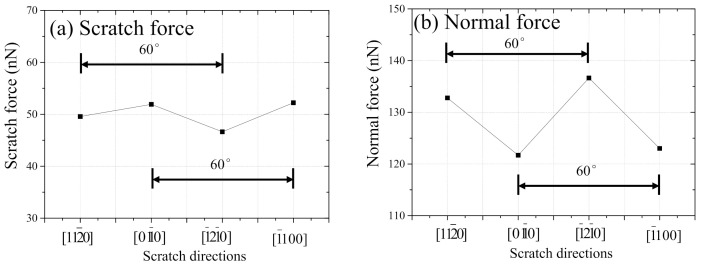
The relationship between scratch direction and (**a**) scratch force and (**b**) normal force, respectively (MD).

Figure 11 shows the relationship between scratch direction and (a) scratch force and (b) normal force, respectively. Both forces were average values from the start to a scratch distance of 8 nm. In Figure 11a, higher scratch forces are obtained when the scratch directions are $\left[01\bar{1}0\right]$ and $\left[\bar{1}100\right]$ than those of $\left[11\bar{2}0\right]$ and $\left[\bar{1}2\bar{1}0\right]$. This trend is the same as that of the experimental results shown in Figure 6a. On the contrary, Figure 11b indicates that higher normal forces are observed when the scratch directions are $\left[11\bar{2}0\right]$ and $\left[\bar{1}2\bar{1}0\right]$ than those of $\left[01\bar{1}0\right]$ and $\left[\bar{1}100\right]$. Higher loads lead to higher hardness or smaller plasticity. This indicates that extra or undesired plastic deformation is unlikely to occur when the scratch directions are $\left[11\bar{2}0\right]$ and $\left[\bar{1}2\bar{1}0\right]$. In both figures, the intervals between adjacent peaks or valleys are 60°. This illustrates that Zn(0001) has a six-fold symmetrical machinability originating from the atomic arrangements in the basal plane just as observed in the aforementioned experimental results of the c-plane sapphire.

### 6.2. Comparison between Experimental and Simulation Results

**Figure 12:**
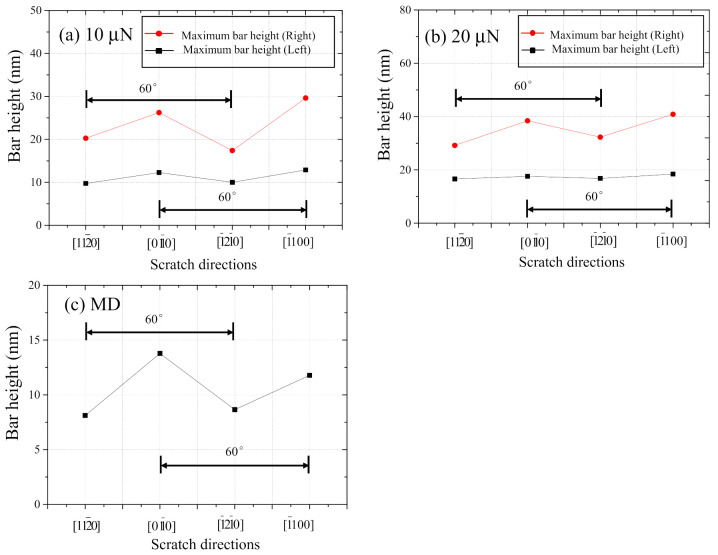
Comparison between experimental ((**a**) 10 μN, (**b**) 20 μN) and (**c**) MD simulation results regarding the effect of scratching direction on burr height.

Figure 12 show the comparison between experimental ((a) 10 µN and (b) 20 µN) and (c) MD simulation results regarding the effect of scratching direction on burr height. In the experimental results shown in Figure 12a,b, the heights of the burrs on the left and right are remarkably different although the apex of the diamond tip is rounded. It is because the diamond tip is a triangular pyramid that side-forward nanoscratch tests were performed in the first place. Even so, it can be confirmed that the burr heights become lower, when the scratch tests are conducted in the direction of basal slip such as $\left[11\bar{2}0\right]$ and $\left[\bar{1}2\bar{1}0\right]$. An identical trend can be confirmed in the MD simulation result as well, as shown in Figure 12c.

**Figure 13:**
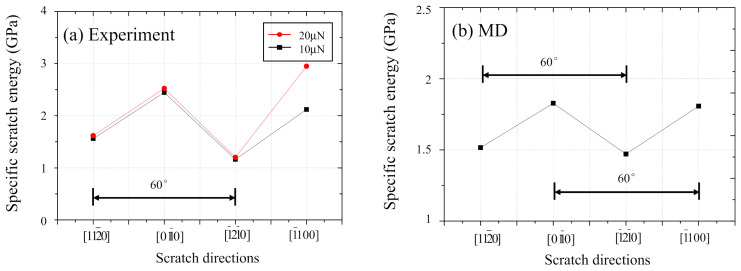
Comparison between (**a**) experimental and (**b**) simulation results regarding the effect of scratching direction on specific scratch energy.

Figure 13 shows the comparison between (a) simulation and (b) experimental results regarding the effect of scratching direction on specific scratch energy. The removal volume in the MD simulation was obtained from the number of Zn atoms moved over one Burgers vector of Zn, and it showed a similar trend to that of the groove depth shown in Figure 6b although not shown here. The specific scratch energy was calculated using a similar method as Equation (1). From the experimental results shown in Figure 13a, when scratched in the direction of basal slip such as $\left[11\bar{2}0\right]$ and $\left[\bar{1}2\bar{1}0\right]$, the specific scratch energies become remarkably lower than those of $\left[01\bar{1}0\right]$ and $\left[\bar{1}100\right]$. An identical trend can obviously be confirmed from the MD simulation result shown in Figure 13b as well.

These results clearly show that the machining efficiency is maximized and the accuracy can be improved by scratching in the basal slip direction. This is due to the larger deformability of basal slip than that of twinning, which would lead to smooth and effective cutting chip formation.

## 7. Conclusions

Single groove nanoscratch experiments were conducted on c-plane sapphire wafer to analyze the ductile-regime removal and deformation mechanism including the anisotropy. As a result, the specific scratch energy showed a trend of six-fold symmetry which was thought to be due to basal slip or twinning. To clarify the nanoscratch mechanism, particularly the anisotropy, an MD simulation of Zn, which is one of the hcp crystals with similar slip/twin systems, was performed. From the comparison between the experimental and the MD simulation results regarding the effect of scratching direction on specific scratch energy and burr height, it was found that in the single groove nanoscratch, the specific scratch energy and the burr height are minimized, and the machining efficiency is maximized while the accuracy can be improved by scratching along the basal slip direction. This is due to the larger deformability of basal slip leading to smooth and effective cutting chip formation.

## Figures and Tables

**Table 1:** Slip/twinning systems and critical shear stresses for sapphire [14,19,21].

Slip/Twinning System	Description	$\mathrm{Critical}\;\mathrm{Shear}\;\mathrm{Stress}\;\tau_{c}(\mathrm{GPa})$
$\left(0001\right)\langle 11\bar{2}0\rangle$	Basal slip	2.7 < $\tau_{c}$ < 5.0
$\left(0001\right)\langle \bar{1}010\rangle$	Basal twinning	$\tau_{c}$ > 4.0
$\left\{11\bar{2}0\right\}\langle \bar{1}100\rangle$	Prism slip	2.3 < $\tau_{c}$ < 5.7
$\left\{0\bar{1}11\right\}\langle 10\bar{1}1\rangle$	Pyramidal slip	$\tau_{c}$ > 7.0
$\left\{\bar{1}012\right\}\langle 10\bar{1}1\rangle$	Rhombohedral twinning	1.0 < $\tau_{c}$ < 8.1

**Table 2:** Representative slip/twin systems for zinc [22].

Slip/Twin System	Description
$\left(0001\right)\langle 11\bar{2}0\rangle$	Basal slip
$\left\{1\bar{1}00\right\}\langle 11\bar{2}0\rangle$	Prismatic slip
$\left\{1\bar{1}01\right\}\langle 11\bar{2}0\rangle$	Pyramidal slip <*a*>
$\left\{11\bar{2}2\right\}\langle \bar{11}23\rangle$	Pyramidal slip <*c*+*a*>
$\left\{10\bar{1}2\right\}\langle \bar{1}011\rangle$	Tensile twin
$\left\{10\bar{1}1\right\}\langle \bar{1}012\rangle$	Compression twin

**Table 3:** Experimental conditions for the single groove nanoscratch test.

Instrument	SPM (SPA-300HV, Seiko Instruments Co., Japan)
Workpiece	Sapphire (c-plane)
Diamond tip	Trigonal pyramid (Tip radius = 100 nm)
Cantilever stiffness (N/m)	200
Normal load for observation (µN)	0.2
Normal load for nanoscratch test (µN)	10, 20
Scratch direction	$\left[11\bar{2}0\right]$, $\left[10\bar{1}0\right]$, $\left[2\bar{11}0\right]$, $\left[\bar{1}100\right]$, $\left[1\bar{2}10\right]$, $\left[0\bar{1}10\right]$, $\left[\bar{11}20\right]$
Scratch speed (µm/s)	16
Scratch length (µm)	5

**Table 4:** Morse potential parameters.

Parameter	Zn-Zn [30]	C-Zn
*D* (eV)	0.1700	0.06418
α (Å)	1.705	2.13
$r_{0}$ (Å^−1^)	2.793	2.6363

**Table 5:** Conditions for MD simulation of single groove nanoscratch.

Workpiece	Monocrystalline Zinc (Zn(0001))
Dimension of analysis area of workpiece (nm)	Radius: 8.1, Height: 6.7
Scratching tool tip	Monocrystalline diamond (C)
Dimension of analysis area of tool tip (nm)	Tip radius: 4.5, Height: 2.2
Initial and thermostat temperatures (K)	300
Scratch depth (nm)	1.3
Scratch length (nm)	8
Scratch speed (m/s)	20
Scratch direction	$\left[11\bar{2}0\right]$,$\left[10\bar{1}0\right]$,$\left[2\bar{11}0\right]$,$\left[\bar{1}100\right]$

## Data Availability

Not applicable.
